# Multi-layer CNN-LSTM network with self-attention mechanism for robust estimation of nonlinear uncertain systems

**DOI:** 10.3389/fnins.2024.1379495

**Published:** 2024-04-04

**Authors:** Lin Liu, Jun Feng, Jiwei Li, Wanxin Chen, Zhizhong Mao, Xiaowei Tan

**Affiliations:** ^1^College of Information Science and Engineering, Northeastern University, Shenyang, Liaoning, China; ^2^State Key Laboratory of Robotics, Shenyang Institute of Automation, Chinese Academy of Sciences, Shenyang, Liaoning, China; ^3^Institutes for Robotics and Intelligent Manufacturing, Chinese Academy of Sciences, Shenyang, Liaoning, China; ^4^University of Chinese Academy of Sciences, Beijing, China

**Keywords:** nonlinear systems, surface electromyography signal, machine learning network, uncertainties, robust estimation

## Abstract

**Introduction:**

With the help of robot technology, intelligent rehabilitation of patients with lower limb motor dysfunction caused by stroke can be realized. A key factor constraining the clinical application of rehabilitation robots is how to realize pattern recognition of human movement intentions by using the surface electromyography (sEMG) sensors to ensure unhindered human-robot interaction.

**Methods:**

A multilayer CNN-LSTM prediction network incorporating the self-attention mechanism (SAM) is proposed, in this paper, which can extract and learn the periodic and trend characteristics of the sEMG signals, and realize the accurate autoregressive prediction of the human motion information. Firstly, the multilayer CNN-LSTM network utilizes the CNN layer for initial feature extraction of data, and the LSTM network is used to improve the enhancement of the historical time-series features. Then, the SAM is used to improve the global feature extraction performance and parallel computation speed of the network.

**Results:**

In comparison with existing test is carried out using actual data from five healthy subjects as well as a clinical hemiplegic patient to verify the superiority and practicality of the proposed algorithm. The results show that most of the model’s prediction *R* > 0.9 for different motion states of healthy subjects; in the experiments oriented to the motion characteristics of patient subjects, the angle prediction results of *R* > 0.99 for the untrained data on the affected side, which proves that our proposed model also has a better effect on the angle prediction of the affected side.

**Discussion:**

The main contribution of this paper is to realize continuous motion estimation of ankle joint for healthy and hemiplegic individuals under non-ideal conditions (weak sEMG signals, muscle fatigue, high muscle tension, etc.), which improves the pattern recognition accuracy and robustness of the sEMG sensor-based system.

## 1 Introduction

Stroke and other diseases may lead to lower limb motor dysfunction in patients. With the assistance of robotic technology, intelligent rehabilitation therapy can be realized to reduce the workload of clinical medical staff and improve the efficiency of patients’ rehabilitation training (Kapelner et al., 2020). In the human–machine interaction between rehabilitation robots and patients, traditional human–machine interaction techniques often involve the robot passively receiving instructions, which may not be convenient for patients with motor function impairments (Zhai et al., 2017; Zhang et al., 2022). In recent years, human–machine interaction technology needs to evolve toward allowing robots to actively understand human behavioral intentions, resulting in a new type of interaction based on human biological signals.

Human bioelectric signal is the potential difference activated when the nerve signal containing human behavioral information is transmitted to the relevant organs or tissues, which is a direct reflection of human behavioral intentions (Ma et al., 2021). It is of great significance to break the human–machine barrier and realize natural human–machine interaction by decoding human bioelectric signals to recognize human behaviors, and empowering robots to understand the human body’s intentions as an information medium for interaction between human beings and the outside world (Qi et al., 2020). Currently, widely studied bioelectric signals include electromyogram (EMG), electroencephalogram (EEG), electrocardiogram (ECG), and electrooculography (EOG). We focus on the surface electromyography (sEMG), which originates from the bioelectrical activity of spinal motor neurons under the control of the motor cortex of the brain, and are the temporal and spatial sum of sequences of action units produced by peripherally active motor units. Since sEMG has the advantages of being non-invasive, and simple to use, it is more suitable to be applied to the design of human–machine interaction control systems for rehabilitation robots (Xiong et al., 2021). The core technology to build the EMG human–machine interaction system is to decode the human body’s motion intention through EMG signals, and the usually discussed motion intention decoding includes two categories, one is to recognize the discrete limb movements based on sEMG, such as the movements of the hand’s clenched fist, extended palm, etc., and the other is to estimate the continuous joint motions based on sEMG, such as the continuous quantities of the joint moments and the joint angles, etc. In this study, we focus on healthy people and hemiplegic patients, and carry out research on sEMG-based continuous motion estimation methods for the foot and ankle area of the lower limb, which lays the foundation for future natural human–machine interaction control.

Human walking characteristics are crucial in studies targeting the continuous movement of the lower limb. Many features of the musculoskeletal system of the lower limbs implied in the human walking information. Human walking information can be used as a basis for the recognition of human movement intentions and the estimation and prediction of the human body’s movements, which in turn improves the stability and accuracy of human–computer interactions with external devices, such as exoskeletons. It is also possible to compare the gait characteristics of different walking bodies, especially between healthy and patients. This enables an intelligent online evaluation of patient rehabilitation effects, such as stroke rehabilitation. Lower limb walking in healthy people is cyclic, and the inherent states of its musculoskeletal system, such as human limb properties and muscle activation states, are also relatively stable and have good model interpretability, so mechanistic models have been used to describe them in many studies (Zhang L. et al., 2021). There are also some research works that describe machine learning models such as neural networks with straightforward modeling process and unrestricted utilization of sEMG.

However, in research focused on hemiplegic patients, there are large differences in the nature of the bilateral cyclic reciprocity, with the healthy side usually experiencing weak functional decline and the affected side experiencing more severe fluctuations in cyclic information (Aymard et al., 2000; Zhao et al., 2023). The alternation of useful and useless information can lead to problems such as gradient disappearance or gradient explosion, causing loss of information (Meng et al., 2023). In addition, these weakly abled people are also prone to problems such as muscle fatigue or even spasticity, and in some cases excessive muscle tone (Zhang et al., 2019; Moniri et al., 2021), all of which will lead to a high degree of difficulty in estimating the continuity of a patient’s lower extremities based on EMG signals (Sarasola-Sanz et al., 2018; Fleming et al., 2021; Zhu et al., 2022).

In machine learning network architectures for the study of continuous lower limb motion, auto-regression is a widely used method for time series prediction. It can capture the correlation and dependency of input and output sequences well, and has the advantages of simple structure, flexible order selection and easy application (Lehtokangas et al., 1996). The observations at the current time of the time series data are correlated with the historical observations. Autoregressive technologies can make use of cyclical, trend and seasonal characteristics of historical data to predict future data (Yin et al., 2023). The combination of autoregressive techniques and neural networks can effectively improve the ability of learning, understanding and forecasting of time series data (Taskaya-Temizel and Casey, 2005). A nonlinear autoregressive neural network with exogenous inputs has been proposed to model the dynamic behavior of an automotive air conditioning system (Ng et al., 2014). Combing autoregressive integrated moving average (ARIMA) and probabilistic neural network (PNN), a hybrid network model has been proposed in order to improve the prediction accuracy of ARIMA models (Khashei et al., 2012). Therefore, this article will process the sampled motion data by autoregressive technology, so that the network can fully learn the hidden features and improve the learning efficiency of the network.

In order to improve the robustness of time series signal prediction, a convolutional neural network (CNN) can be used to extract initial features from the data (Shao et al., 2024). The CNN is a specific type of feedforward neural network with a grid topology (Li Z. et al., 2021). CNN uses sparse interaction, parameter sharing and variant representation techniques to improve the feature extraction performance of convolutional operations (Li et al., 2016). Each convolution layer of CNN contains multiple convolution kernels, and each convolution checks data for sliding convolution to achieve feature extraction of time series data to obtain local features and short-term dependencies. The pooling layer performs summary statistics on the output obtained by the convolution layer (Gu et al., 2018). The local perception and weight sharing of CNN can also effectively reduce the number of weight parameters for model learning, thus improving the efficiency of model learning. Based on deep CNN, a joint multi-task learning algorithm has been developed to predict effectively attributes in images (Abdulnabi et al., 2015). A joint classification-and-prediction framework has been proposed based on CNN for automatic sleep staging (Phan et al., 2018). Combing CNN architecture with depth wise separable convolutions with kernels (CNN-DSCK) has developed for prediction rating exploiting product review (Khan and Niu, 2021). The prediction applications of these complex systems show the advantages of CNN networks in time series feature extraction. For complex and long-term dynamic systems, whose data series have long-term correlation, LSTM network with better long-term feature capture ability can be considered for feature extraction (Bi et al., 2021; Zhang N. et al., 2021; Zha et al., 2022). LSTM network is an improvement of recurrent neural network (RNN) network, which can effectively improve the gradient disappearance and gradient explosion of RNN network in time series prediction (Kim and Cho, 2019). Complex system prediction based on LSTM network has achieved a series of innovative results (Rathore and Harsha, 2022). Based on multi-layer LSTM networks, a forecasting method with a strong capability has been proposed for predicting highly fluctuating demand (Abbasimehr et al., 2020). According to the characteristics of chemical process data, a key alarm variables prediction model has been developed in chemical process based on dynamic-inner principal component analysis (DiPCA) and LSTM network (Bai et al., 2023). Adding self-attention mechanism after LSTM network can further capture the correlation between features directly from a global perspective (Zhang et al., 2020). Increasing attention mechanisms can also compensate for gradient disappearance or gradient explosion problems that LSTM networks face, which can lead to loss of information in time series data (Li J. et al., 2021). By integrating CNN, attention mechanism and LSTM, it is expected to build a network with better predictive performance.

Therefore, this article proposes a robust multi-layer network with excellent performance by integrating LSTM network with CNN network and adding self-attention mechanism technology. In order to extract and learn the period and trend characteristics of EMG signals, autoregressive processing is performed on the collected data. The CNN layer is used to extract the features from the EMG signal. The LSTM network is used to consolidate and enhance the historical temporal features. self-attention mechanism (SAM) is utilized to improve the global feature extraction performance and the parallel computing speed of the network. Finally, compared with the existing algorithm, the superiority and practicability of the proposed network are verified by using the data of healthy laboratory subjects and clinical patients with hemiplegia.

The main contributions of this article are as follows: (1) To address the periodicity of human lower limb gait walking, a multi-layer machine learning network architecture has been designed. It improves the interpretability and prediction accuracy of the auto-regression model, and reduces the problems of gradient disappearance or explosion caused by redundant sensor information. (2) The practicality of the algorithm has been validated, undergoing testing not only on healthy individuals but also utilizing data from hemiplegic patients. It has successfully achieved continuous lower limb motion estimation under non-ideal conditions (weak sEMG signals, muscle fatigue, high muscle tension, etc.). This ensures both accuracy and robustness in identification, laying a foundation for the design of human–machine interaction methods for future rehabilitation robots.

In order to facilitate understanding, the chapter part of this article is summarized as: a novel artificial intelligence algorithm is proposed in section “2 Materials and methods,” the experiments and results are presented in section “3 Experiments and results,” and finally main key conclusions of this article are given in section “4 Discussion and conclusion.”

## 2 Materials and methods

### 2.1 Data acquisition and processing

**FIGURE 1:**
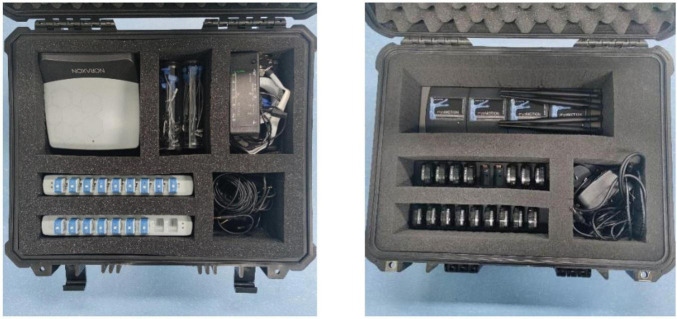
Noraxon sEMG and inertial sensor acquisition system.

**FIGURE 2:**
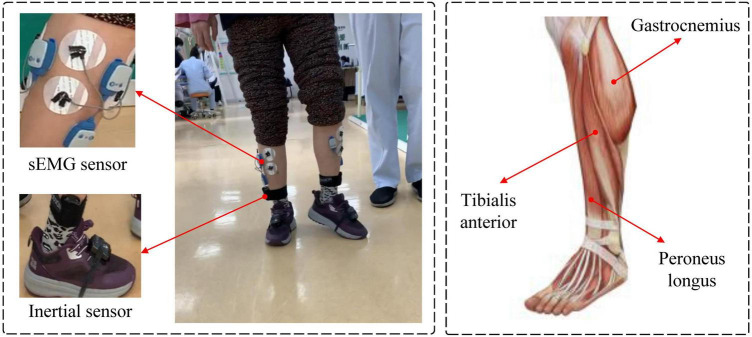
Setup of the EMG signal acquisition experiment.

Five subjects (age: 26.6 ± 2.6 years, height: 1.74 ± 0.08 m, weight: 69 ± 10.9 kg) and one patient tester (male, 67 years old, Brunnstrom stage IV) participated in the data collection of this experiment. The sEMG signal acquisition equipment is a Noraxon Ultium EMG system and AgCl electrodes, as shown in Figure 1. Alcohol wipes are used to wipe the surface skin of the tested muscles to remove impurities such as dead skin and sweat adhering to the skin surface. Two electrodes for each channel are spaced 20 mm apart and affixed to the muscle belly along the muscle fiber direction of the target muscles of both legs of the subjects (Hermens et al., 2000). Subjects walk on a treadmill at 2.0 km/h, 3 km/h, and 5.0 km/h and EMG signals are collected. Subjects walk for 3 min at a time with a 1-min rest between each trial to avoid the effects of muscle fatigue. The sEMG sampling frequency is 1,200 Hz, as shown in Figure 2, three muscles of the ankle joint, tibialis anterior, peroneus longus, and gastrocnemius are collected. Meanwhile, the kinematic parameters are collected using a Noraxon myoMOTION Inertial Measurement Unit (IMU), which collects the angular changes in the sagittal plane of the ankle joint of the lower limb, with a sampling frequency of 200 Hz. Written informed consent was signed by all subjects before inclusion in this study. The experimental procedures follow the Declaration of Helsinki and were approved by the Ethics Committee of Liaoning Provincial People’s Hospital (Grant No. 2022HS007).

In this experiment, the ankle EMG input and output signals of five healthy individuals and one stroke patient are used as test and validation signals for the network model. Four sets of test data with a length of 120,000 are obtained from the left foot of the healthy tester under four states: 2 km/h speed, 3 km/h speed, 5 km/h speed, and plantarflexion dorsiflexion maneuver. Two tests are conducted on the left foot of the stroke patient and the length of the sampled data is taken as 70,000. Due to the large amount of noise signals in the original acquired sEMG signals, and the frequency range of sEMG signals are in the range of 0–500 Hz. In this article, the original sEMG signals are filtered and denoised, and then the irrelevant noises are removed from the original sEMG signals in order to retain the valuable information as much as possible. The sEMG signals are first band-pass filtered with a fourth-order 10–500 Hz Butterworth band-pass filter. Then, a 50 Hz trap filter is used to eliminate the industrial frequency interference. After that, the data used will be normalized.

### 2.2 CNN-LSTM networks with self-attention

#### 2.2.1 Convolutional neural network

For the collected data, CNN uses convolution layer to convolve the input vector matrix to extract the local features of the time series data. The feature sequence generation equation is shown in Equation 1.


(1) $$C_{h\,i}=f\,\left(W_{h}\,X_{i:i+h-1}+b\right)$$

where *W_h_* is the weight matrix of the convolution kernel; *b* is biased unit; *X*_*i:i+h–*1_ is the sequence matrix from *i* to *i* + *h* − 1 in a time series; *h* is the size of the convolution kernel; *f* is the activation function.

The calculated feature set *C_n_* can be expressed as Equation 2.


(2) $$C_{n}=\left\{C_{1},C_{2},\ldots ,C_{i+h-1}\right\}$$

The pooling layer extracts the features of the time series obtained by the convolution layer, outputs a matrix of fixed size, reduces the dimension of the output result and retains the features. In this article, the maximum pooling method is used to calculate the pooling layer. The computational equation of the eigenvector after the pooling of convolution nuclei is represented by Equation 3.


(3) $$C_{p\,o\,o\,l}=M\,a\,x\,\left(C_{1},C_{2},\ldots ,C_{n-h+1}\right)$$

#### 2.2.2 LSTM neural network

LSTM network is a variant of RNN. The key point of LSTM is to control the flow and forgetting of information through the use of structures called gates. The function of these gates is to selectively allow information to pass through or prevent the flow of information, and the core unit is the cell state, which can be regarded as the network’s memory. The LSTM network consists of several key components.

1. Cell state: it is the main storage unit of LSTM and is responsible for storing and transmitting information.
2. Input gate: the input gate determines whether new information is added to the status unit at the current time step.
3. Forget gate: the forget gate determines what information is deleted from the state unit.
4. Output gate: the output gate determines which information in the state unit is output to the next time step. The relevant calculation formulas are shown as Equations 4–9.

(4) $$I_{t}=\sigma \,\left(X_{t}\,W_{x\,i}+H_{t-1}\,W_{h\,i}+b_{i}\right)$$

(5) $$F_{t}=\sigma \,\left(X_{t}\,W_{x\,f}+H_{t-1}\,W_{h\,f}+b_{f}\right)$$

(6) $$O_{t}=\sigma \,\left(X_{t}\,W_{x\,o}+H_{t-1}\,W_{h\,o}+b_{o}\right)$$

(7) $${\tilde{C}}_{t}=t\,a\,n\,h\,\left(X_{t}\,W_{x\,c}+H_{t-1}\,W_{h\,c}+b_{c}\right)$$

(8) $$C_{t}=F_{t}⊙C_{t-1}+I_{t}⊙{\tilde{C}}_{t}$$

(9) $$H_{t}=O_{t}⊙t\,a\,n\,h\,\left(C_{t}\right)$$

The principle is to combine the current input *X_t_* and the hidden state *H*_*t–*1_ of the previous time step, which are activated by sigmoid function respectively. Calculate the activation value *I_t_* of the input gate; calculate the activation value *F_t_* of the forgetting gate and *O_t_* of the output gate; *X_t_* and *H*_*t–*1_ are combined, and then activated by tanh function to selectively retain the current memory, which is recorded as ${\tilde{C}}_{t}$; the state *C*_*t–*1_ of the previous time step is selectively forgotten by using the forgetting gate *F_t_*. The input gate *I_t_* is used to selectively retain the current state ${\tilde{C}}_{t}$ of the time step, and the two are added together to update the state unit *C_t_*. Multiply the new state unit *C_t_* with the output gate *O_t_* to get the hidden state *H_t_* of the current time step.

#### 2.2.3 Self-attention mechanism

Compared with conventional networks such as RNN and LSTM, which process the features of time series data with equal weight, self-attention can calculate the correlation degree between each time series data from a global perspective, and allocate different attention to different locations at the end to enhance the main features of time series data. The self-attention mechanism can flexibly adapt to different input sequences and task requirements. For time series *A* = [*a*_1_, *a*_2_, …*a*_*n*_], the attention *B* = [*b*_1_, *b*_2_, …*b*_*n*_] of each position is obtained by obtaining the correlation degree between the sequence data. The specific calculation process is as follows.

Each value of the sequence *A* maps to three different spaces. For each input *a_i_*, multiply by three trainable weights *w_q_*, *w_k_*, and *w_v_*, respectively, to obtain three values of *q_i_*, *k_i_*, and *v_i_*, namely query, key, and value as shown in Equations 10–12.


(10) $$q_{i}=w_{q}\cdot a_{i}$$

(11) $$k_{i}=w_{k}\cdot a_{i}$$

(12) $$v_{i}=w_{v}\cdot a_{i}$$

Using the weight matrices, *W_q_*, *W_k_*, and *W_v_*, they can be further expressed in the following matrix form, as shown in Equations 13–15.


(13) $$Q=W_{q}\cdot A$$

(14) $$K=W_{K}\cdot A$$

(15) $$V=W_{V}\cdot A$$

**FIGURE 3:**
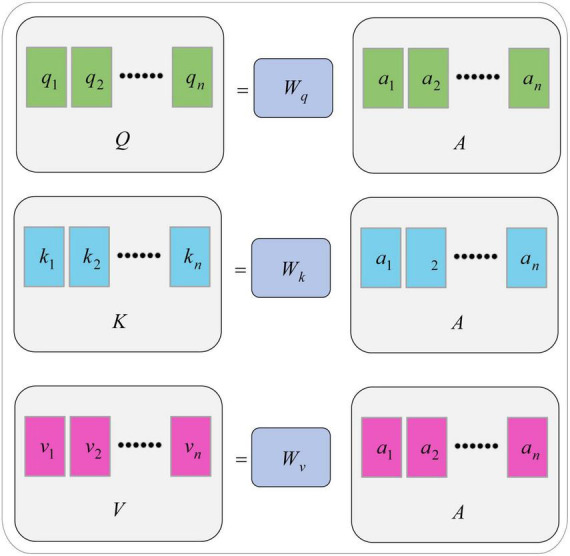
Generation of *Q*, *K* and *V* matrices.

The generation diagram of matrix *Q*, *K*, and *V* is shown in Figure 3.

With each input value *a*_*i*_(*i* = 1, …*n*) corresponding to *q_i_*, and all input values *a_j_* corresponding to *k_j_*, calculate the degree of correlation between *a_i_* and *a_j_* by dot product, as shown in Equation 16.


(16) $$\delta_{i,j}={\left(k_{i}\right)}^{\tau }\cdot q_{j}$$

Its matrix form is shown in Equation 17.


(17) $$\Delta =K^{T}\cdot Q$$

Dividing δ_*i*,*j*_ by the dimension $\sqrt{d_{k}}$ of *q_i_* or *k_i_* can control the size of the dot product result to prevent situations where the gradient is too large or too small and leads to poor training results, as shown in Equation 18.


(18) $$\alpha_{i,j}=\frac{\delta_{i,j}}{\sqrt{d_{k}}}$$

Its matrix form is shown in Equation 19.


(19) $$\Lambda =\frac{\Delta }{\sqrt{d_{k}}}$$

The activated correlation matrix Λ′ can be obtained by softmax operation on the correlation matrix Λ.

**FIGURE 4:**
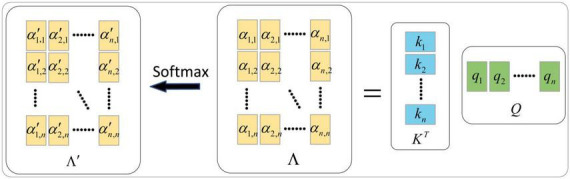
Generation of Λ and Λ′.

The calculation process is shown in Figure 4.

Use the resulting Λ′ and *V* to calculate the attention corresponding to each input vector *a_i_* as shown in Equation 20.


(20) bi=∑j=1nvj⋅α′i,j

Its matrix form can be expressed as Equation 21.


(21) $$B=V\cdot \Lambda^{\prime }$$

where *B* is the matrix of attention *b_i_*.

The computational equation of the self-attention mechanism can be summarized as Equation 22.


(22) $$O\,u\,t\,p\,u\,t=s\,o\,f\,t\,m\,a\,x\,\left(\frac{K^{T}\,Q}{\sqrt{d_{k}}}\right)\,V$$

**FIGURE 5:**
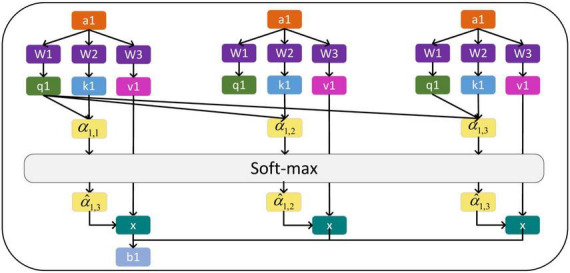
Generation of output matrix *O*.

The calculation process of attention *b*_1_ for the first input value *a*_1_ is shown in Figure 5.

#### 2.2.4 CNN-LSTM network

**FIGURE 6:**
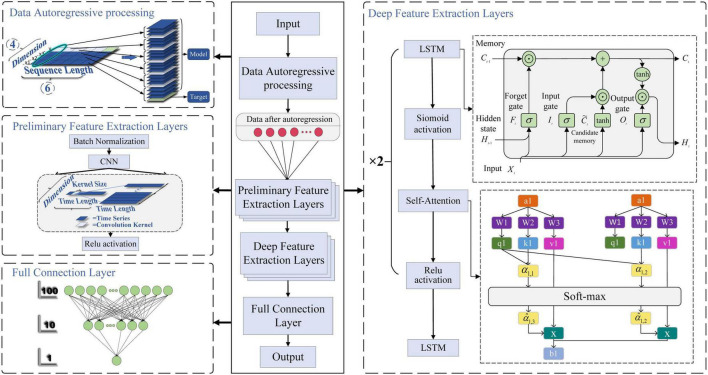
Proposed CNN-LSTM network model with self-attention mechanism.

The proposed CNN-LSTM prediction model integrated with self-attention mechanism in this article is shown in Figure 6. The predictive network model mainly includes data autoregressive processing, preliminary feature extraction layer based on CNN network, depth feature extraction layer based on LSTM network, and full connection layer. Note that in practical engineering applications, the collected data should be cleaned reasonably, including removing singular values, averaging and noise elimination which can effectively improve the training and testing effect of the network. The time step of autoregression cannot be taken too long or too short. If the time step is taken too long, the less relevant time series information in the past may be added to the current information prediction, which may reduce the prediction accuracy. If the time step is taken too short, it may reduce the correlation extraction between continuous data. Therefore, in the practical application process, the regression time step should be selected according to the specific research object and sequence characteristics. When the network is used for online prediction or control, too many network layers may improve the prediction accuracy of the algorithm, but it may also increase the computing burden of the network.

##### 2.2.4.1 Autoregressive processing

Surface electromyography has typical nonlinear and fast time-varying characteristics, and it is difficult to capture and extract the trend of sEMG by conventional fitting methods. In order to improve the periodicity, trend and seasonality of the output data, autoregressive processing should be carried out on the pre-training data.

##### 2.2.4.2 Preliminary feature extraction layer

In order to make the input data after autoregressive processing easier to train, the batch normalization layer (BN layer) is used to normalize each batch. The normalized operation of the BN layer can not only improve the convergence speed of the network model, but also enhance the correlation degree between the data in the batch, and prevent the model from overtraining some data and resulting in overfitting. Then, the features of the time series are initially extracted by using CNN. The CNN can not only extract features through the convolution operation of multiple convolution kernels, but also obtain local dependencies of sequence data by convolution operation with sliding window. For the obtained features, the ReLU activation layer can be activated to enhance the expression ability of the features.

##### 2.2.4.3 Depth feature extraction layer

The extracted features can be used to further extract the long-term dependencies in the time series data through the LSTM network. The data processed by the LSTM layer enters the Sigmoid layer for activation. Then, the self-attention mechanism is added to calculate the correlation between all features and the weight matrix, and the weight matrix is constantly trained, so that the model can allocate attention independently according to the data characteristics, and improve the role of features in prediction. Finally, the ReLU activation layer is used to activate the features. After the above two deep feature extraction, the obtained deep features are fed into the LSTM layer for comprehensive strengthening and consolidation.

##### 2.2.4.4 Fully connected layer

The features obtained from the depth feature extraction layer are mapped to the fully connected layer to obtain the prediction results. In order to make the model have stronger generalization ability and avoid the problem of gradient vanishing or gradient explosion, the network proposed in this article adopts the strategy of gradually decreasing the number of neurons, and uses two linear mapping layers in the fully connected layer to continuously reduce the number of neurons, and obtains the single-valued prediction result. In addition, adding the intermediate mapping layer can also enable the model to learn more feature combinations and representations.

## 3 Experiments and results

### 3.1 Performance evaluation

The *R*^2^ score and Root Mean Square Error (*RMSE*) are commonly used as evaluation metrics of regression performance for continuous estimation of joint angles (Zhong et al., 2022). In order to obtain more accurate continuous estimation results for the lower extremity joints, the regression performance of the lower extremity hip, knee, and ankle joints is evaluated using the following *R*^2^ performance metrics. *R*^2^ and *RMSE* are defined as shown in Equations 23, 24, respectively:


(23) $$R^{2}=1-\frac{\overset{\overset{i=1}{n}}{\Sigma }{\left(\theta_{i}-\hat{\theta_{i}}\right)}^{2}}{\overset{\overset{i=1}{n}}{\Sigma }{\left(\theta_{i}-\bar{\theta }\right)}^{2}}\in \left[0,1\right]$$

(24) $$R\,M\,S\,E=\sqrt{\frac{\overset{\overset{i=1}{n}}{\Sigma }{\left(\theta_{i}-{\hat{\theta }}_{i}\right)}^{2}}{n}}$$

where θ_*i*_ is the actual value of the angle of the target joint, ${\hat{\theta }}_{i}$ is the angle of the joint predicted by the model, $\bar{\theta }$ is the average value of the actual angle θ_*i*_, and *n* is the length of the sampling sequence. In addition, we perform a statistical analysis using one-way analysis of variance (ANOVA) under the 0.05 level of significance.

### 3.2 Result and discussion

**FIGURE 7:**
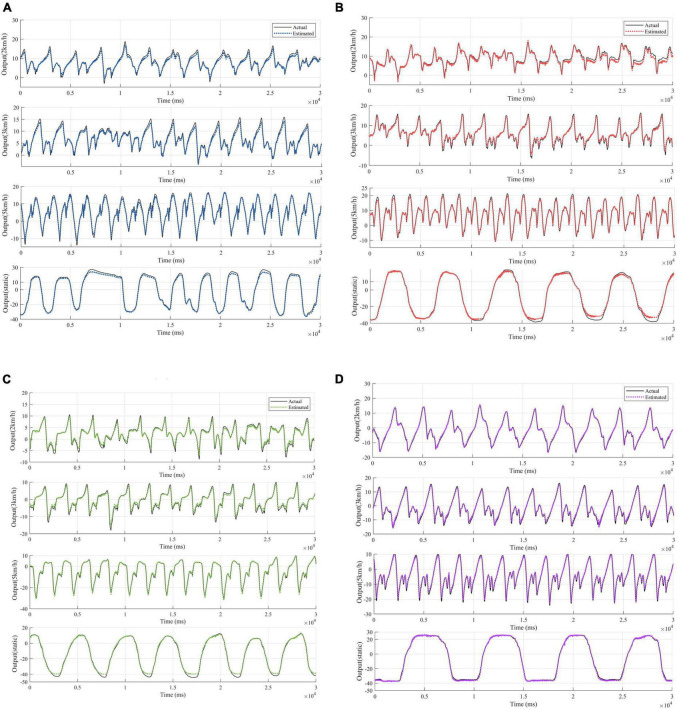
Fitted curves of ankle EMG signals at four exercise speeds in four healthy subjects. **(A)** Fitting results for subject 1. **(B)** Fitting results for subject 2. **(C)** Fitting results for subject 3. **(D)** Fitting results for subject 4.

**FIGURE 8:**
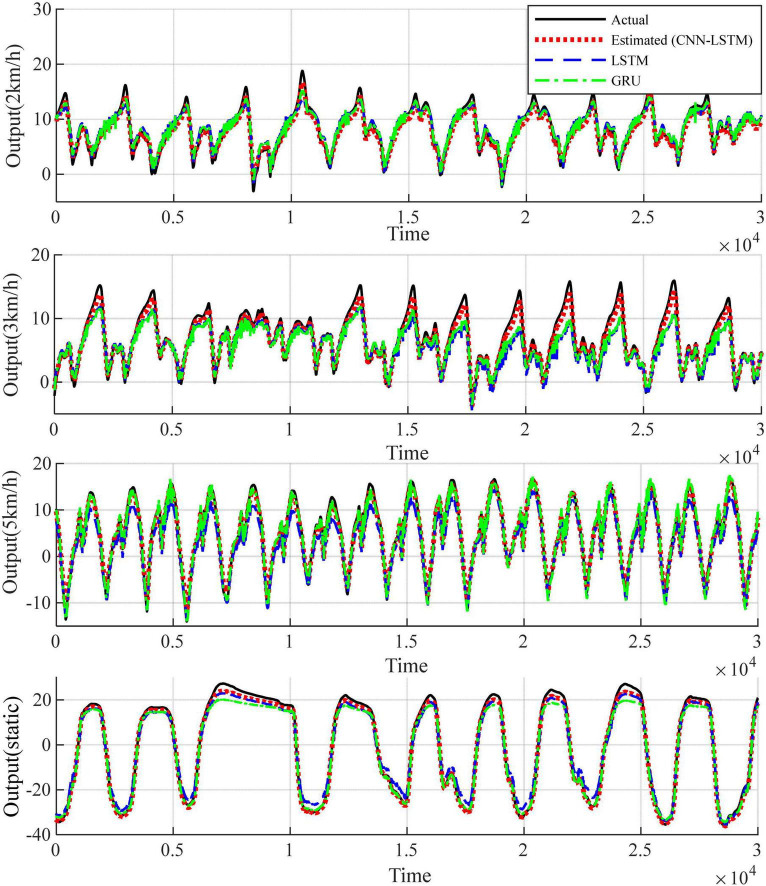
Comparison of the fitting results of the proposed algorithm for healthy subject 1 with the experimental results of the existing algorithm.

**FIGURE 9:**
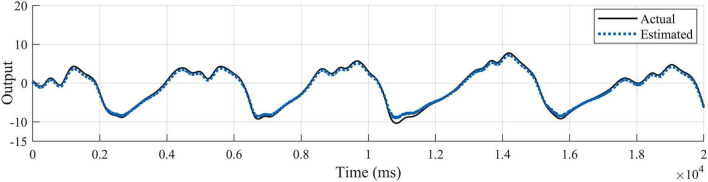
Fitted curves of EMG signals of the healthy ankle from the first set of data of the diseased test subjects.

Since each healthy person has 4 test states, 5 healthy people contain 20 sets of test data. The patients contain 2 sets of test data. Considering that the output signals are characterized by obvious periodicity, trend and seasonality, autoregressive processing is performed on the test data in order to highlight the characteristics of the output data for the training of the proposed network model. According to the length of the data sequence, the data of the first 4 healthy subjects, which is the first 90,000 points of the 16 sets of data with the first 50,000 points of the first set of data of the patients are taken respectively. The time step of autoregression is chosen as 5, which is the input and output data at the moment of *t*–1, *t*–2,……*t*–5 and the input data at the moment of *t* are used simultaneously for the prediction of the output data at the moment of *t*. In the experimental process, for the proposed network, one layer CNN is set for initial feature extraction. A two-layer LSTM network with SAM followed by one-layer LSTM is used for depth feature extraction. Three full connection layers is applied to obtain the predicted output. Adam is used as an optimizer to determine the optimal solution of the loss function. The parameter setting strategy of the network not only ensures that the network captures data characteristics efficiently, but also does not have too much computational burden. Since the data input dimension is 3 and the output dimension is 1, the data before combination is a vector of 6 rows and 4 columns. The data after regression combination is transformed into a vector of 1 row and 24 columns, where the data in the first 23 columns are treated as input data to the model, and the data in the last 1 column is the output data corresponding to the current t moment. Combined with the time dimension, the length of the time series is 1,490,000. The first 23 columns of data are input into the model to obtain the predicted value *y* of the model, and the loss value is calculated for the predicted value and the 24th column of output data in order to update the parameters of the network model and complete the learning and training of the model. Using the trained model, predictions are made for the posterior 30,000 points of data for the first 4 health testers, and for the posterior 20,000 points of data for patient group 1. The test results for the four groups of health testers are shown in Figure 7. In order to highlight the superiority, the existing prediction LSTM algorithm in Dong et al. (2023) and the algorithm in Zhou et al. (2022) are also tested for prediction healthy subject 1 in Figure 8. Comparison and verification results show that the proposed prediction algorithm has better prediction accuracy and robustness. The results for patient group 1 are shown in Figure 9. The results show that the proposed method has good tracking performance for lower limb motion angle prediction.

The results of the MSE comparison between the predicted data and the real data for the different exercise modes are shown in Table 1. It can be seen from the table that the MSE errors in the walking condition are small and within 1° for both healthy and diseased subjects. The static metatarsal dorsiflexion exercise had an MSE within 5° due to the wider angular range of the exercise. *R*^2^ can be used as a better criterion for use in this study as the error range of MSE fluctuates due to the different angular ranges of motion of different subjects in different modes of motion.

**TABLE 1:** Comparison of MSE based on training data.

	Healthy (group 1)	Healthy (group 2)	Healthy (group 3)	Healthy (group 4)	Patient (group 1)
2 km/h	0.7412	0.6723	0.5221	0.2811	0.2362
3 km/h	0.5030	0.6530	0.9210	0.6909	\
5 km/h	0.6857	0.6110	0.6741	0.6267	\
Static	2.4677	3.7219	3.1074	1.0042	\

#### 3.2.1 Effects of different exercise modes

The ankle angle prediction curves for the four movement speeds of the testers are shown in Figures 7, 9. Not only the tracking deviation between the estimated angle and the target angle of healthy subjects is small in the CNN-LSTM model, but the model also shows good regression performance for the ankle motion of diseased testers. It can be seen that our proposed multilayer CNN-LSTM network model incorporating the self-attention mechanism has good tracking performance and high prediction accuracy.

**FIGURE 10:**
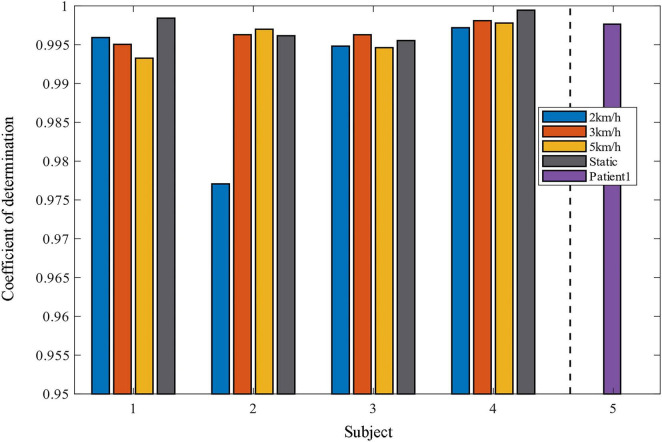
Histogram of the coefficient of determination of projected versus real data.

**FIGURE 11:**
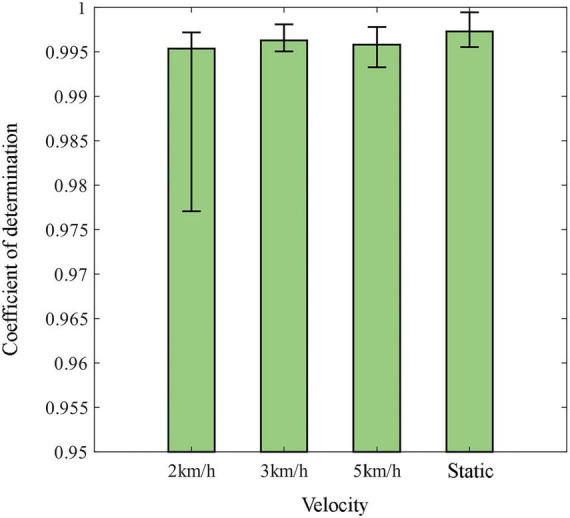
Comparison of the coefficient of determination between predicted and real data for different exercise modes.

Two indicators, *RMSE* and *R*^2^ are used to evaluate the quality of the method in predicting the ankle joint angle. Comparison of the coefficient of determination between the predicted data and the real data under different motion modes is shown in Figures 10, 11, and it can be seen that most of the coefficients of determination are above 0.99, which means that the predicted data of the model have a better correlation with the real data, and they can reflect the dynamic characteristics of the system very well. It should be noted that the movement of the tester at 2 km/h belong to slow movement. Due to the difference in height and weight, in this case, the lower limbs of the test subjects cannot be fully moved, resulting in different EMG signals for each test subject. The test results in Figure 7 also show that the prediction error is larger under the motion state of 2 km/h. Therefore, the error band for 2 km/h is larger in Figure 11. When the walking speed is increased to 3 km/h, it is closer to the natural walking speed of the human body, the human gait is more natural, the muscle coordination is stable and flexible, and the prediction performance will be improved. However, when the walking speed increases to 5 km/h, which is faster than the human walking speed, the accuracy starts to decrease. This indicates that the closer the walking speed is to the normal walking speed of human body, the better the muscle coordination of the lower limbs is; when it is faster or slower than the normal walking speed of human body, the muscles of the lower limbs are in a situation of insufficient coordination or fatigue, which is contrary to the normal pattern, and the prediction results of the model will decline more and more, which is in line with the normal walking law. For the static plantarflexion state, the model predicted the best results, which may be due to the fact that there is no floor force contact with the ankle plantar dorsiflexion in the static state, which reduces the complex interactions between the foot and the ground or the influence of shoes.

#### 3.2.2 Effects between healthy and patient subjects

The MSE values of healthy subjects and patients under walking exercise showed significant differences. Since people with weak abilities are also prone to problems such as muscle fatigue or even cramps, and in some cases there are factors such as high muscle tone, this study can further explore the reasons for these differences, such as physical fitness differences, testing conditions, and health status. It helps to understand the differences in physiological responses between healthy and sick subjects at different exercise intensities. As shown in Table 1 the MSE values of sick subjects are generally lower than those of healthy subjects, which suggests that there are some differences between the results of sick testers and the expected values in these particular exercises.

#### 3.2.3 Effects of model-oriented motor characteristics of healthy and patient subjects

For patients with lower limb motor dysfunction, there is usually a more severe motor deficit on the affected side, whereas the functional decline is usually weaker on the healthy side. As a result, the sEMG signals we acquire often alternate between useful and useless information, which can lead to problems such as gradient vanishing or gradient explosion, causing loss of information. In addition, these dysfunctional people are prone to problems such as muscle fatigue and even spasticity. The angles of their lower limb movements are more complicated and abnormal. Therefore, extracting the relationship between sEMG signals and movement trajectories under the above more complicated and non-ideal factors is a challenging problem.

**FIGURE 12:**
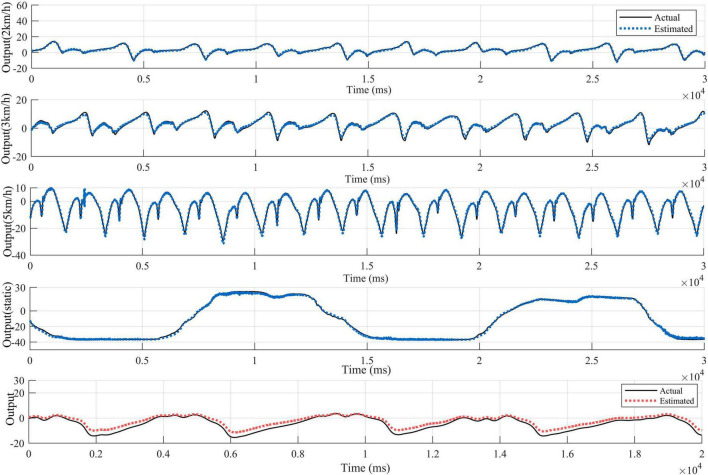
Cross-validation of ankle EMG signal fitting curves for the fifth healthy tester and the second set of data from the diseased tester.

**FIGURE 13:**
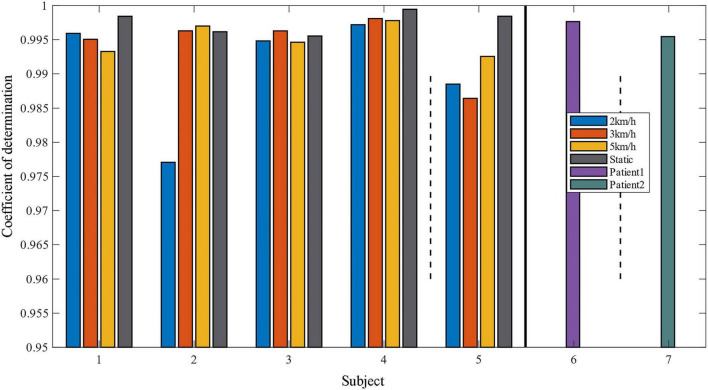
Comparison of determination coefficients between prediction and cross-validation data.

In order to verify the generalization performance of our proposed model and the motion estimation performance for subjects with lower limb motor dysfunction, we input the second set of untrained data from the fifth healthy and diseased participants into the model for ankle joint angle prediction. The results are shown in Figure 12. For healthy subject 5, who is not included in the trained dataset, sEMG and corresponding movement angles are measured at different movement speeds (2 km/h, 3 km/h, 5 km/h) and static plantarflexion and dorsiflexion movement states. The results in Figure 13 indicate that, in comparison with subjects 1–4, the predicted *R* for the walking angle of healthy subject 5 without model training is slightly lower but still greater than 0.985. However, with *p* > 0.05, the difference is not statistically significant. This confirms that the model exhibits good prediction performance for data from subjects not included in the training set. For patient subject 2, the *R* > 0.99 for the untrained data on the affected side demonstrates the effectiveness of our proposed model in predicting angles on the affected side. However, as indicated in Table 2, the MSE value is higher compared to the training data, reaching 6.0589. This may be attributed to the extensive angle fluctuation in the late stages of exercise due to muscle spasm or fatigue in diseased subjects. Consequently, the MSE value of the prediction exhibits a substantial error compared to that of the training data.

**TABLE 2:** Comparison of MSE based on test data.

2 km/h (healthy group 5)	3 km/h (healthy group 5)	5 km/h (healthy group 5)	Static (healthy group 5)	Left foot (patient group 2)
0.2728	0.6909	0.6267	1.0042	6.0589

By estimating the lower limb motion for subjects other than the training data, it can be seen that our proposed method not only has better model generalization ability, but also can predict the lower limb motion angle of the patient in a more ideal way. This helps to identify the physiological differences between healthy individuals and patients in specific movement states or static states. Further analysis can try to identify the physiological or medical factors that lead to these differences, which has potential applications for disease diagnosis, treatment, or health status assessment.

## 4 Discussion and conclusion

In this article, a multilayer CNN-LSTM prediction network model incorporating a self-attention mechanism is proposed. In order to validate the performance of the model in predictive tracking of ankle joint mobility for different populations. The remaining data of both healthy and patient subjects are treated as test data and inputted into the model, and the prediction results of different motion states for the fused model are compared. The results show that most of the model’s prediction *R* > 0.9 for different motion states of healthy subjects; in the experiments oriented to the motion characteristics of patient subjects, the angle prediction results of *R* > 0.99 for the untrained data on the affected side, which proves that our proposed model also has a better effect on the angle prediction of the affected side. Therefore, the model we propose in this article not only has a good exercise estimation ability for healthy subjects, but also can be used for exercise estimation of lower limb dysfunction, which helps to understand the differences in physiological responses between healthy and patients under different exercise modalities, and further analysis can try to find out the physiological or medical factors that lead to these differences, which can then be used for the evaluation of rehabilitation efficacy oriented to clinical patients. The main merits of the proposed method include that the design network architecture has been designed and improves the interpretability and prediction accuracy of the auto-regression model, and reduces the problems of gradient disappearance or explosion caused by redundant sensor information.

Electromyogram neural information collected from the human body provides a new idea for human–robot interaction, and this study provides a feasible solution for accurately estimating the ankle angle of the lower extremity in both health and patients. Future work can be applied to the control of exoskeleton robots, clinical rehabilitation training and evaluation. However, this study also has some limitations. Since patients with post-stroke hemiparesis are virtually unable to perform lower limb walking movements in Brunnstrom stage I and II, our experiment was only able to estimate movements for patients in stage III and above. Subsequently, we will expand the number and range of subjects and explore multi-sensor fusion methods to enhance the reliability of the model.

## Data availability statement

The raw data supporting the conclusions of this article will be made available by the authors, without undue reservation.

## Ethics statement

The studies involving humans were approved by the Ethics Committee of Liaoning Provincial People’s Hospital. The studies were conducted in accordance with the local legislation and institutional requirements. The participants provided their written informed consent to participate in this study. Written informed consent was obtained from the individual(s) for the publication of any potentially identifiable images or data included in this article.

## Author contributions

LL: Conceptualization, Investigation, Methodology, Validation, Writing – original draft. JF: Data curation, Formal analysis, Software, Writing – original draft. JL: Conceptualization, Methodology, Validation, Writing – review & editing. WC: Data curation, Formal analysis, Software, Writing – original draft. ZM: Funding acquisition, Project administration, Resources, Writing – review & editing. XT: Funding acquisition, Project administration, Resources, Writing – review & editing.
